# Determination of physical and mechanical properties of carrot in order to reduce waste during harvesting and post‐harvesting

**DOI:** 10.1002/fsn3.760

**Published:** 2018-08-22

**Authors:** Ahmad Jahanbakhshi, Yousef Abbaspour‐Gilandeh, Tarahom Mesri Gundoshmian

**Affiliations:** ^1^ Department of Biosystems Engineering University of Mohaghegh Ardabili Ardabil Iran

**Keywords:** carrots, harvest, mechanical properties, physical properties, postharvest operations, waste control

## Abstract

Lack of sufficient knowledge about the physical and mechanical properties of agricultural products can result in higher waste of them. Due to the importance of carrot as an agricultural product and lack of much knowledge about how to reduce its waste as well as design and optimize the required harvest and postharvest machinery, this research study was carried out to fill this gap. In this study, physical properties included the length, width, thickness, mean diameter (geometric and arithmetic), mass, volume, density, sphericity, surface area, aspect ratio. The mechanical properties of the samples and their lengths were measured under the conditions of pressure (bruise), bending (break), and shearing of the carrot halves using a Zwick/Roell Instron testing machine based on the recommended standards. The mean geometric mean diameter, surface area, sphericity, volume and true density of the carrot were 49.54 mm, 7758.32 mm^2^, 0.32%, 70 cm^3^, and 1.04 g/cm^3^. In the study of mechanical properties of carrots, the maximum forces required for bruising, bending, and shearing of the carrot fruit were 71.90, 48.60, and 41.14 N, respectively. The results obtained about the physical and mechanical properties can be very useful in reducing carrot waste and mechanizing harvest and postharvest operations by providing us with information that helps us design machinery needed to transfer, sort, separate, wash, package, store, and process carrots.

## 1 INTRODUCTION

Carrot is one of the most important agricultural products used by millions of people all over the world. The scientific name of it is *Daucus carota* L. which belongs to the family of Umbelliferae. Carrot is one of the most important and useful vegetables for the human body since it contains nutrients and vitamins. Eating carrots increases an individual's resistance to infectious diseases. Carrots are used in raw, cooked, juice, and pickled forms (Abbas, 2017; Erenturk & Erenturk, 2007).

Some of the waste in agricultural products at different stages such as harvest, transfer, transportation, and processing is caused by unexpected loads and stresses upon them. Moreover, in order to process agricultural products, some loading needs to be done through cuts in or pressure on the product. Thus, to prevent mechanical harm and waste during the harvest processes and the stages after that and to optimize processing devices, it is necessary to measure and study physical and mechanical properties of agricultural products (Akbarnejad, Azadbakht, & Asghari, 2017; Vishwakarma, Shivhare, & Nanda, 2012). During the processes of transfer and storage, fruits get bruised due to the pressure on them that is caused by heavy loads. Such damages reduce the quality of the product and increase the waste rate (Akbarnejad et al., 2017; Jaliliantabar, Lorestani, Gholami, Behzadi, & Fereidoni, 2011; Rong, Qunying, & Deqiang, 2004). Processing and proper transportation techniques for agricultural products require precise data regarding the physical properties of them such as the shape, size, porosity, surface area, and density (Jahanbakhshi, Yeganeh, & Akhoundzadeh Yamchi, 2016; Topuz, Topakci, Canakci, Akinci, & Ozdemir, 2005). Density and porosity affect the structural loads and are considered as important parameters in designing storage and drying systems (Mpotokwane, Gaditlhatlhelwe, Sebaka, & Jideani, 2008). Stiffness parameters can be used as indexes for the vulnerability of the agricultural products. Weight, volume, density, and average geometric diameter are the factors used to describe agricultural products (Goyal, Kingsly, Kumar, & Walia, 2007; Jahanbakhshi et al., 2016).

Rasekh and Majdi (2012) studied the mechanical properties of garlic. They reported that the forces necessary to loosen the garlic bulbs when loading them along the height and in the lateral yield were 127.023 to 228.001 N and 45.52 to 106.97 N respectively. Kılıçkan and Güner (2008) studied physical and mechanical properties of olive fruit under compressive loading. They showed that the special deformation and rupture energy changes significantly increased as with increase in size and deformation rate and the highest values of change took place along the longitudinal dimension. Rasekh (2014) investigated some mechanical properties of black‐eyed peas and reported that moisture had a significant effect on all mechanical properties at 1% probability level. In addition, he stated that increase in moisture would increase the amount of the energy required for rupture, toughness and deformation at the point of rupture increased while the required force for rupture decreased.

Numerous studies have been carried out on the physical and mechanical properties of different products such as banana (Akbarnejad et al., 2017), tomato (Reddy & Srinivas, 2017), crabapple (Altuntaş, 2015), scolymus (Jahanbakhshi et al., 2016), pomegranate (Jithender, Vyas, Abhisha, & Rathod, 2017), and cherry (Çalışır & Aydın, 2004) so far.

The aim of this study is to determine some mechanical properties of carrot fruit. The results of the research will be very useful in designing different devices in the processes of production to supply the product with reduced waste and damage.

## 2 MATERIALS AND METHODS

### 2.1 Determination of physical properties

In this study, the carrots which were at the full ripeness level were collected. Then, any external material and premature or damaged carrots were taken away. To prevent the product from losing its initial moisture, the samples were kept in a refrigerator at the temperature of 4 ± 1°C and about two hours before the experiments. To reach the room temperature, the samples transferred from the storage space (the refrigerator) to the laboratory. To do so, 20 g carrot samples were placed inside the oven for four hours at the temperature of 105°C in three replications (Doymaz, 2007; Jahanbakhshi et al., 2016). To measure the weight of the samples before and after being placed in the oven, a digital scale (GF600, USA model) with the accuracy of 0.01 g was used. Then, the moisture content and dry matter of carrot fruit were calculated by Equations (1) and (2), respectively (Moghadam & Kheiralipour, 2015):

(1) $$\text{MC}=\frac{M_{w}-M_{d}}{M_{w}}\times 100,$$

(2) $$\text{DM}=\frac{M_{d}}{M_{w}}\times 100,$$

where, MC is the moisture content of fruit (%), *M*_w_ is the initial mass of fruit (g), *M*_d_ is the mass of dried fruit (g), and DM is the dry matter fruit (%).

The means for the moisture content and the dry matter of carrot fruit were 87.18% and 12.82%. The properties of carrots were measured at their initial natural moisture. To measure physical properties, 100 samples of carrot were selected randomly. Dimensions of the carrot (their length (*L*), width (*W*), and thickness (*T*)) were measured using a DC‐515, Taiwan digital caliper with the precision of 0.01 mm. Then, the geometric mean diameter, arithmetic mean diameter, and sphericity were calculated through Equations 3, 4, and 5.

(3) $$D_{g}=\sqrt[{3}]{L.W.T.},$$

(4) $$D_{a}=\frac{L\;+\;W\;+\;T}{3},$$

(5) $$Ø=\frac{D_{g}}{L},$$

*D*_g_ is the geometric mean diameter, *D*_a_ is the arithmetic mean diameter and Ø is the sphericity of the carrot. The surface area (*S*) and the aspect ratio (*R*_a_) of the carrot were obtained through Equations 6 and 7.

(6) $$S=\pi D_{g}^{2},$$

(7) $$R_{a}=\frac{W}{L}.$$

The carrots mass was measured using a GF600, USA digital scale with the precision of 0.01 g. To determine the volume of the carrots, the platform method was used (Mohsenin, 1986). Thus, the samples were dipped in a beaker that was placed on a scale using a legged clamp. The second reading of the scale showed the weight of the fruit dipped into the water minus the weight of the container and the water which equaled the weight of the displaced water. This weight is inserted into the following and the volume of the carrots is calculated through Equation 8.

(8) $$V=\frac{W_{w}}{\rho_{w}}$$

The density of the carrots is obtained by Equation 9.

(9) $$\rho_{t}=\frac{M}{V}$$

where *W*_w_ is the density of the displaced water (g/cm^3^), *ρ*_W_ is the density of the water (g/cm^3^), ρ_t_ is the true density (g/cm^3^), *M* is the mass (g), and *V* is the volume of the carrots (m^3^).

### 2.2 Determination of mechanical properties

**Figure 1:**
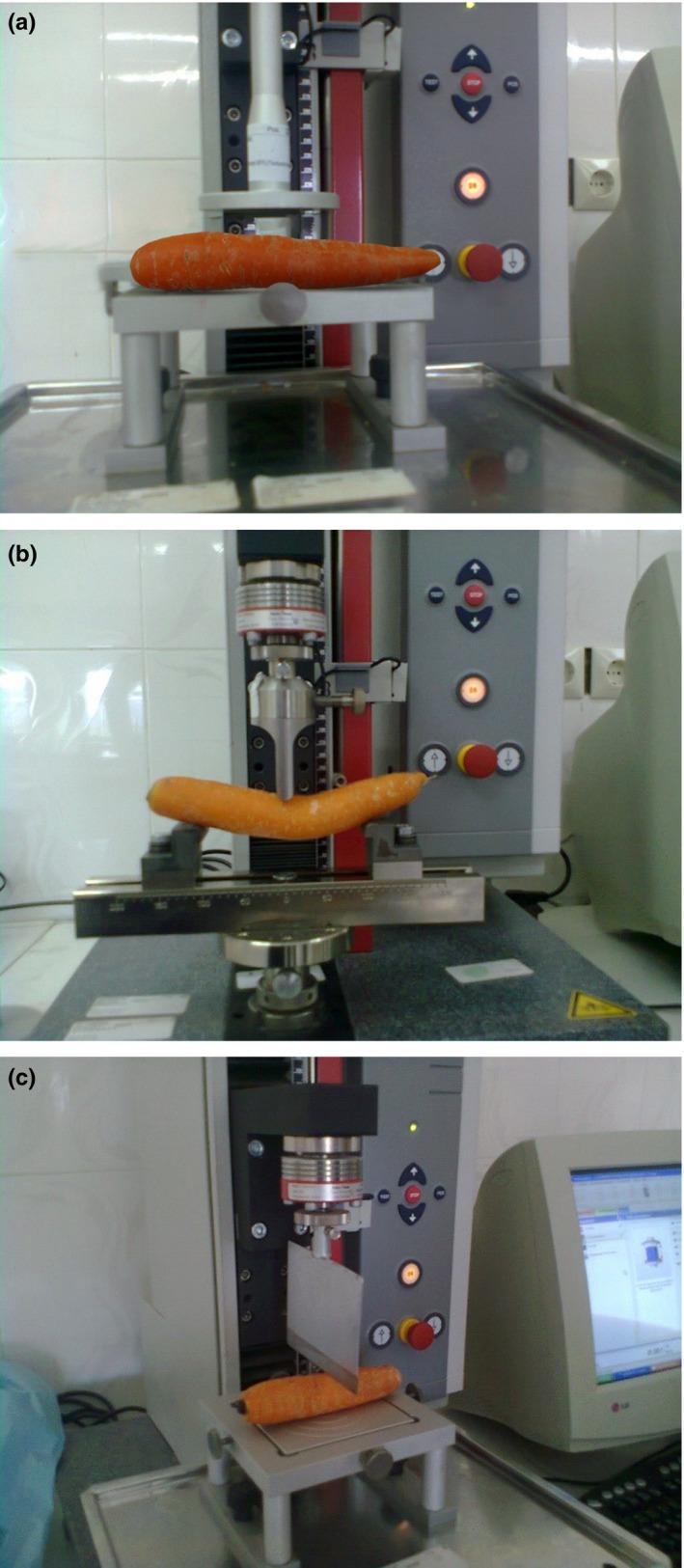
(a) Bruise test, (b) break test, and (c) shear test

In order to determine the mechanical properties of the carrot fruit three tests of pressure (bruise), break (bending), and shear were performed. To do so, Zwick/Roell Instron machine (Z 0.5 model) was used. To test bruising, a round pressure prop and to test bending, a three‐point prop were used under vertical loading based on the ASTMD 790‐03 standard and for the shear test, a straight edge blade with the thickness of 1.4 mm and the blade angle of 30° was used based on the DIN 53294 standard at room temperature at the testing speed of 20 mm/min (Jahanbakhshi, 2018; Jahanbakhshi et al., 2016; Jaliliantabar et al., 2011). The Instron machine was simultaneously connected to a computer and data mining was carried out. An example of the graphs, related to the bruise, bending and shearing tests, is shown in Figure 1.

## 3 RESULTS AND DISCUSSION

### 3.1 Physical properties

The amounts physical properties of carrots are shown in Table 1. The mean length, width, thickness, arithmetic mean diameter, geometric mean diameter, surface area, sphericity, aspect ratio, mass, volume, and True density of the carrot were 154.55, 28.61, 27.60, 70.49, 49.54 mm, 7758.32 mm^2^, 0.32%, 0.18, 72.74 g, 70 cm^3^, 1.04 g/cm^3^. Agricultural products’ wastes are partly due to improper packaging and insufficient transportation equipment. Packaging must take into account the requirements of transportation and marketing in terms of the weight, size and shape of the agricultural products. The information obtained in this research can be used in this regard. Since the length of the carrot has a great difference with its width and thickness, it can be concluded that carrot has a low sphericity (0.32%) and this property must be taken into consideration in designing transfer, handling, and grading systems. The true density of the carrot was 1.04 (g/cm^3^) and this property can be used in designing the transfer systems, displacement, and sorting. The volume and area of the product are applied in modeling matter and heat transfer during the drying or freezing processes. In similar research, Jahanbakhshi (2018), Jaliliantabar et al. (2011), Karaj and Müller (2010) and Akar and Aydin (2005) has discussed and stressed upon the importance of these properties in determining the size of the machines particularly that of the separation, transfer, and sorting equipment.

**Table 1:** Physical properties of carrot fruit

Parameter	Mean	Max	Min	*SD*	CV %
Length (mm)	154.55	185	121.03	17.61	11.39
Width (mm)	28.61	33.50	23.06	2.45	8.56
Thickness (mm)	27.60	32.50	22	2.63	9.52
Arithmetic mean diameter (mm)	70.49	78.49	63.39	4.30	6.10
Geometric mean diameter (mm)	49.54	58.10	40.96	4.12	8.31
Surface area (mm^2^)	7,758.32	10,599.58	5,270.22	1,273.84	16.41
Sphericity (%)	0.32	0.38	0.28	0.02	6.25
Aspect ratio	0.18	0.24	0.15	0.02	11.11
Mass (g)	72.74	115	44.95	16.73	22.99
Volume (cm^3^)	70	112	43	16.31	23.30
True density (g/cm^3^)	1.04	1.17	1	0.03	2.88

### 3.2 Mechanical properties

Table 2 shows the mechanical properties of carrot in the pressure (bruise) test. The mean values of the properties measured in the pressure test (elasticity module, the maximum force required to bruise the carrot fruit, deformation occurrence as the maximum force acting, the work done to reach the maximum force required for bruise the carrot fruit under vertical load) were 0.038 MPa, 71.90 N, 19.32 mm, and 862.32 N.mm respectively. Fruit tissue hardness is one of the parameters that can be affected by static and dynamic loads and fruit quality might decrease as a result of change in hardness. Modulus of elasticity is one of the parameters that can be used to measure fruit tissue hardness. The higher the modulus of elasticity is, the harder the fruit tissue will be. In a similar study, Jahanbakhshi (2018) examined the mechanical properties of snake melon fruit. In a bruise test, they reported the modulus of elasticity equal to 0.027 MPa. Compared to the present study, it can be stated that carrot fruit has a higher modulus of elasticity and thus has a tighter tissue. Agricultural products often show different behaviors regarding their strength in front of pressure forces. In order to minimize mechanical damage, the pressure forces caused during the transfer of the products must be reduced to the minimum rate (lower than 71.90 N).

**Table 2:** Mechanical properties of carrot fruit in the pressure test

Parameters	Mean	Max	Min	*SD*	CV %
Elasticity modulus (MPa)	0.038	0.046	0.029	0.007	18.42
*F*_max_ (N)	71.90	92.30	56.70	17.88	24.86
DL at *F*_max_ (mm)	19.32	21.70	17.40	1.60	8.28
W to *F*_max_ (N.mm)	862.32	935.20	789.80	52.06	6.03

The mechanical properties of the carrot fruit in the break (bending) test are shown in Table 3. The mean values of the measured properties in the bending test (elasticity module, the maximum force required to bend the carrot fruit, deformation occurred at maximum value of bending force, the required work to reach the maximum force required to bend the carrot fruit under vertical load) were 0.027 MPa, 48.60 N, 21.90 mm, and 504.52 N.mm respectively. The results indicate that in order to prevent carrot fruit from breaking in the packaging, transportation, and processing, the application of forces above the value of 48.60 N must be avoided.

**Table 3:** Mechanical properties of carrot fruit in the bending test

Parameter	Mean	Max	Min	*SD*	CV %
Elasticity modulus (MPa)	0.027	0.031	0.024	0.002	7.40
*F*_max_ (N)	48.60	50.20	45.30	1.92	3.95
DL at *F*_max_ (mm)	21.90	26.70	18.50	3.52	16.07
W to *F*_max_ (N.mm)	504.52	577.20	428.80	52.98	10.50

The mechanical properties of the carrot fruit in the shear test are shown in Table 4. The mean values of the measured properties in the shearing test (shear modulus, maximum force required for shearing carrot fruit, shear strength, and shear deformation when applying maximum force) were 0.014 N/mm^2^, 41.14 N, 0.0017 N/mm^2^, and 11.82 mm respectively. The data obtained about the mechanical properties through the shear test can be applied in the carrot fruit processing factories. In study, Jahanbakhshi et al. (2016) applied a shear test to examine the mechanical properties of scolymus and reported the average shear modulus of 0.002 N/mm^2^. Comparing the result of that study with the present one, it was concluded that the carrot fruit has a high shear modulus of 0.014 N/mm^2^, hence this product has a high resistance against shear strain. Jahanbakhshi (2018) reported the mean value of the required force for shearing the snake melon was 33.66 N. The higher shear force of 41.14 N required for shearing carrot indicate a high resistance of the carrot fruit to shear forces.

**Table 4:** Mechanical properties of carrot fruit in the shear test

Parameters	Mean	Max	Min	*SD*	CV %
Shear modulus (N/mm^2^)	0.014	0.016	0.013	0.001	7.14
*F*_B_ (N)	41.14	44.40	37.80	2.40	5.83
*T*_B_ (N/mm^2^)	0.0017	0.0018	0.0016	0.00007	4.11
ν_B_ (mm)	11.82	12.80	10.80	0.74	6.26

## 4 CONCLUSION

1. In addition to the importance of studying physical and mechanical properties in minimizing mechanical damage, these properties are considered as the basic data in designing the machinery and equipment used during the harvesting and in the postharvesting operations.
2. In examining mechanical properties, the maximum force required in bruising, bending and shearing tests of the carrot fruit were 71.90, 48.60, and 41.14 N respectively.
3. In comparison to the shear module obtained in previous studies, the high shear module for carrot fruit in this study (0.00324 N/mm^2^) shows its high resistance against shear strain.

## CONFLICT OF INTEREST

The authors have declared no conflict of interest.

## ETHICAL REVIEW

This study does not involve any human or animal testing.
